# 3,4,6-Trimethyl-1-phenyl-1*H*-pyrazolo­[3,4-*b*]pyridine

**DOI:** 10.1107/S1600536810026474

**Published:** 2010-07-10

**Authors:** Salha Hamri, Abderrafia Hafid, Hafid Zouihri, Saïd Lazar, Mostafa Khouili

**Affiliations:** a Laboratoire de Chimie Organique et Analytique, Equipe COOA, Faculté des Sciences et Techniques, Université Sultan Moulay Slimane, BP 523, 23000 Beni-Mellal, Morocco; b Laboratoires de Diffraction des Rayons X, Centre Nationale pour la Recherche Scientifique et Technique, Rabat, Morocco; c Laboratoire de Biochimie, Environnement et Agroalimentaire (URAC 36), Faculté des Sciences et Techniques Mohammedia, Université Hassan II Mohammedia-Casablana, BP 146, 20800 Mohammedia, Morocco

## Abstract

In the title compound, C_15_H_15_N_3_, the 1*H*-pyrazolo­[3,4-*b*]pyridine system and the phenyl ring are each individually planar, with r.m.s. deviations of 0.017 (2) and 0.011 (2) Å, respectively; the dihedral angle between the two aromatic systems is 9.33 (10)°. The crystal packing is stabilized by offset π–π stacking between parallel pyrazolo­[3,4-*b*]pyridine ring systems [face-to-face distance = 3.449 (6) Å].

## Related literature

For a general review of pyrazolo­pyridines, see: Hardy (1984 ▶). For related compounds displaying biological activity, see: Chu & Lynchj (1975 ▶). For bond-length data, see: Allen *et al.* (1987 ▶).
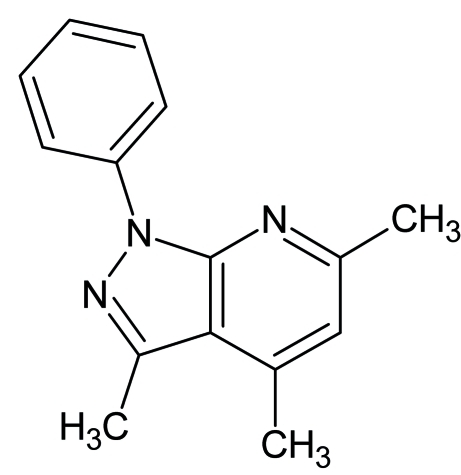


## Experimental

### 

#### Crystal data

C_15_H_15_N_3_
*M* *_r_* = 237.30
Monoclinic, 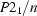

*a* = 7.1714 (2) Å
*b* = 12.0690 (4) Å
*c* = 14.5491 (5) Å
β = 101.251 (1)°
*V* = 1235.05 (7) Å^3^
*Z* = 4
Mo *K*α radiation
μ = 0.08 mm^−1^
*T* = 296 K
0.32 × 0.29 × 0.12 mm

#### Data collection

Bruker X8 APEXII CCD area-detector diffractometer
10642 measured reflections
2252 independent reflections
1841 reflections with *I* > 2σ(*I*)
*R*_int_ = 0.029

#### Refinement

*R*[*F* ^2^ > 2σ(*F* ^2^)] = 0.068
*wR*(*F* ^2^) = 0.198
*S* = 1.09
2252 reflections
166 parameters
H-atom parameters constrained
Δρ_max_ = 0.74 e Å^−3^
Δρ_min_ = −0.26 e Å^−3^

### 

Data collection: *APEX2* (Bruker, 2005 ▶); cell refinement: *SAINT* (Bruker, 2005 ▶); data reduction: *SAINT*; program(s) used to solve structure: *SHELXS97* (Sheldrick, 2008 ▶); program(s) used to refine structure: *SHELXL97* (Sheldrick, 2008 ▶); molecular graphics: *PLATON* (Spek, 2009 ▶); software used to prepare material for publication: *publCIF* (Westrip, 2010 ▶).

## Supplementary Material

Crystal structure: contains datablocks I, global. DOI: 10.1107/S1600536810026474/xu2793sup1.cif

Structure factors: contains datablocks I. DOI: 10.1107/S1600536810026474/xu2793Isup2.hkl

Additional supplementary materials:  crystallographic information; 3D view; checkCIF report

## Figures and Tables

**Fig. 1.**
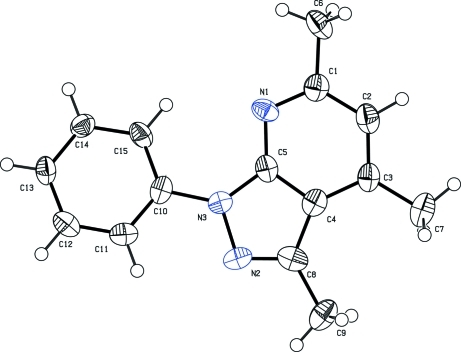
Molecular structure of the title compound with the atom-labelling scheme. Displacement ellipsoids are drawn at the 50% probability level. H atoms are represented as small spheres of arbitrary radii.

## supplementary crystallographic information

### Comment

Many polysubstituted derivatives of 1 *H*-pyrazolo[3,4-*b*]pyridine have been synthesized as potentially biologically active materials (Hardy, 1984; Chu & Lynchj, 1975).

The dihedral angle between the two aromatic ring systems in the title compound, C_15_H_15_N_3_, is 9.33 (10)°. The 1*H*-pyrazolo[3,4-*b*]pyridine and the phenyl rings are planarswith r.m.s. deviation of 0.017 (2) and 0.011 (2) Å, respectively.

The Bond lengths and angles in title compound (Fig. 1) are found to have normal values [Allen *et al.*, 1987]. The crystal packing is stabilized by offset π-π stacking between parallel pyrazolo[3,4-b]pyridine ring sestems related by an inversion center at 1.0, 0.5, 0.0, the face-to-face distance is 3.449 (6) Å.

### Experimental

To a solution of 4-hydroxy-6-methylpyran-2-one (291 mg, 2.309 mmol) and 5-amino-3-methyl-1-phenylpyrazole (200 mg, 1.154 mmol) in 10 ml of *n*-butanol was added *p*-toluenesulfonic acid (0.12 mg).

The reaction mixture was refluxed for 42 h. After evaporation of solvent, the residue was then purified over silica gel column chromatography using a (98:2) mixture of hexane and ethyl acetate as eluent. Under these conditions the compound was obtained as colourless crystals.

### Refinement

All H atoms were fixed geometrically and treated as riding with C—H = 0.96 Å (methyl) and C—H = 0.93 Å (aromatic), *U*_iso_(H) = 1.5*U*_eq_(C) for methyl and *U*_iso_(H) = 1.2*U*_eq_(C) for the others.

### Crystal data

**Table:**

C_15_H_15_N_3_	*F*(000) = 504
*M**_r_* = 237.30	*D*_x_ = 1.276 Mg m^−^^3^
Monoclinic, *P*2_1_/*n*	Mo *K*α radiation, λ = 0.71073 Å
Hall symbol: -P 2yn	Cell parameters from 1348 reflections
*a* = 7.1714 (2) Å	θ = 2.6–25.5°
*b* = 12.0690 (4) Å	µ = 0.08 mm^−^^1^
*c* = 14.5491 (5) Å	*T* = 296 K
β = 101.251 (1)°	Prism, colourless
*V* = 1235.05 (7) Å^3^	0.32 × 0.29 × 0.12 mm
*Z* = 4	

### Data collection

**Table:**

Bruker X8 APEXII CCD area-detector diffractometer	1841 reflections with *I* > 2σ(*I*)
Radiation source: fine-focus sealed tube	*R*_int_ = 0.029
graphite	θ_max_ = 25.3°, θ_min_ = 2.9°
φ and ω scans	*h* = −7→8
10642 measured reflections	*k* = −14→14
2252 independent reflections	*l* = −17→17

### Refinement

**Table:**

Refinement on *F*^2^	Primary atom site location: structure-invariant direct methods
Least-squares matrix: full	Secondary atom site location: difference Fourier map
*R*[*F*^2^ > 2σ(*F*^2^)] = 0.068	Hydrogen site location: inferred from neighbouring sites
*wR*(*F*^2^) = 0.198	H-atom parameters constrained
*S* = 1.09	*w* = 1/[σ^2^(*F*_o_^2^) + (0.104*P*)^2^ + 1.0638*P*] where *P* = (*F*_o_^2^ + 2*F*_c_^2^)/3
2252 reflections	(Δ/σ)_max_ < 0.001
166 parameters	Δρ_max_ = 0.74 e Å^−^^3^
0 restraints	Δρ_min_ = −0.26 e Å^−^^3^

### Special details

**Table:**

Geometry. All e.s.d.'s (except the e.s.d. in the dihedral angle between two l.s. planes) are estimated using the full covariance matrix. The cell e.s.d.'s are taken into account individually in the estimation of e.s.d.'s in distances, angles and torsion angles; correlations between e.s.d.'s in cell parameters are only used when they are defined by crystal symmetry. An approximate (isotropic) treatment of cell e.s.d.'s is used for estimating e.s.d.'s involving l.s. planes.
Refinement. Refinement of *F*^2^ against ALL reflections. The weighted *R*-factor *wR* and goodness of fit *S* are based on *F*^2^, conventional *R*-factors *R* are based on *F*, with *F* set to zero for negative *F*^2^. The threshold expression of *F*^2^ > σ(*F*^2^) is used only for calculating *R*-factors(gt) *etc*. and is not relevant to the choice of reflections for refinement. *R*-factors based on *F*^2^ are statistically about twice as large as those based on *F*, and *R*- factors based on ALL data will be even larger.

### Fractional atomic coordinates and isotropic or equivalent isotropic displacement parameters (Å^2^)

**Table:**

	*x*	*y*	*z*	*U*_iso_*/*U*_eq_	
C1	0.8454 (3)	0.7445 (2)	−0.04712 (18)	0.0367 (6)	
C2	0.7992 (4)	0.6928 (2)	−0.13595 (19)	0.0421 (7)	
C3	0.7465 (3)	0.5818 (2)	−0.14607 (17)	0.0332 (6)	
C4	0.7468 (3)	0.5266 (2)	−0.06073 (18)	0.0351 (6)	
C5	0.7917 (3)	0.5850 (2)	0.02285 (17)	0.0316 (6)	
C6	0.9021 (4)	0.8635 (2)	−0.0425 (2)	0.0472 (7)	
C7	0.6952 (4)	0.5279 (3)	−0.23948 (19)	0.0492 (8)	
C8	0.7099 (3)	0.4171 (2)	−0.0326 (2)	0.0380 (7)	
C9	0.6593 (4)	0.3137 (3)	−0.0909 (2)	0.0481 (8)	
C10	0.8125 (3)	0.5303 (2)	0.19304 (17)	0.0317 (6)	
C11	0.8164 (3)	0.4380 (2)	0.24944 (19)	0.0378 (6)	
C12	0.8416 (4)	0.4501 (2)	0.3447 (2)	0.0404 (7)	
C13	0.8649 (4)	0.5527 (2)	0.38535 (18)	0.0362 (6)	
C14	0.8657 (4)	0.6457 (2)	0.33032 (18)	0.0356 (6)	
C15	0.8396 (3)	0.6356 (2)	0.23326 (18)	0.0355 (6)	
N1	0.8420 (3)	0.69504 (17)	0.03365 (15)	0.0339 (5)	
N2	0.7298 (3)	0.41044 (18)	0.05772 (16)	0.0380 (6)	
N3	0.7801 (3)	0.51439 (17)	0.09427 (15)	0.0332 (5)	
H11	0.8019	0.3677	0.2228	0.045*	
H12	0.8428	0.3877	0.3823	0.048*	
H13	0.8802	0.5597	0.4501	0.043*	
H14	0.8838	0.7153	0.3581	0.043*	
H15	0.8401	0.6980	0.1958	0.043*	
H2	0.8042	0.7343	−0.1893	0.051*	
H6A	0.7931	0.9085	−0.0665	0.071*	
H6B	0.9975	0.8750	−0.0796	0.071*	
H6C	0.9523	0.8835	0.0214	0.071*	
H7A	0.7068	0.5806	−0.2874	0.074*	
H7B	0.5666	0.5016	−0.2488	0.074*	
H7C	0.7792	0.4666	−0.2425	0.074*	
H9A	0.7662	0.2913	−0.1173	0.072*	
H9B	0.5529	0.3288	−0.1405	0.072*	
H9C	0.6268	0.2554	−0.0520	0.072*	

### Atomic displacement parameters (Å^2^)

**Table:**

	*U*^11^	*U*^22^	*U*^33^	*U*^12^	*U*^13^	*U*^23^
C1	0.0326 (13)	0.0418 (15)	0.0371 (14)	0.0062 (11)	0.0100 (11)	−0.0020 (11)
C2	0.0459 (15)	0.0491 (17)	0.0334 (15)	0.0081 (12)	0.0129 (12)	0.0066 (12)
C3	0.0279 (12)	0.0443 (15)	0.0284 (13)	0.0109 (10)	0.0075 (10)	0.0000 (11)
C4	0.0274 (12)	0.0418 (15)	0.0358 (14)	0.0058 (10)	0.0055 (10)	−0.0037 (11)
C5	0.0251 (11)	0.0406 (14)	0.0302 (13)	0.0069 (10)	0.0082 (9)	0.0044 (10)
C6	0.0545 (17)	0.0469 (17)	0.0440 (17)	0.0002 (13)	0.0191 (13)	0.0156 (13)
C7	0.0453 (16)	0.068 (2)	0.0342 (15)	0.0045 (14)	0.0065 (12)	−0.0091 (14)
C8	0.0257 (12)	0.0334 (14)	0.0554 (18)	0.0045 (10)	0.0092 (11)	0.0069 (12)
C9	0.0431 (15)	0.0554 (19)	0.0420 (17)	0.0041 (13)	−0.0011 (12)	−0.0138 (13)
C10	0.0224 (11)	0.0444 (15)	0.0279 (12)	0.0048 (10)	0.0041 (9)	−0.0043 (11)
C11	0.0367 (13)	0.0302 (13)	0.0470 (16)	−0.0026 (10)	0.0096 (11)	−0.0034 (11)
C12	0.0430 (14)	0.0353 (15)	0.0445 (16)	0.0020 (11)	0.0127 (12)	0.0116 (12)
C13	0.0400 (14)	0.0445 (15)	0.0238 (12)	0.0081 (11)	0.0050 (10)	0.0046 (11)
C14	0.0422 (14)	0.0296 (13)	0.0338 (14)	0.0040 (11)	0.0044 (11)	−0.0049 (10)
C15	0.0394 (13)	0.0338 (13)	0.0333 (14)	0.0079 (11)	0.0072 (10)	0.0114 (11)
N1	0.0322 (11)	0.0275 (11)	0.0444 (13)	0.0041 (8)	0.0134 (9)	0.0057 (9)
N2	0.0374 (12)	0.0295 (12)	0.0460 (14)	−0.0025 (9)	0.0053 (10)	−0.0084 (10)
N3	0.0373 (11)	0.0284 (11)	0.0339 (12)	−0.0010 (9)	0.0068 (9)	−0.0026 (9)

### Geometric parameters (Å, °)

**Table:**

C1—C6	1.491 (4)	C10—C11	1.380 (4)
C2—H2	0.9300	C11—H11	0.9300
C2—C1	1.415 (4)	C11—C12	1.370 (4)
C3—C7	1.487 (4)	C12—H12	0.9300
C3—C4	1.408 (4)	C13—H13	0.9300
C3—C2	1.392 (4)	C13—C14	1.379 (4)
C4—C8	1.424 (4)	C13—C12	1.368 (4)
C4—C5	1.388 (4)	C14—H14	0.9300
C6—H6C	0.9600	C15—H15	0.9300
C6—H6B	0.9600	C15—C10	1.397 (4)
C6—H6A	0.9600	C15—C14	1.393 (4)
C7—H7C	0.9600	N1—C5	1.377 (3)
C7—H7B	0.9600	N1—C1	1.322 (3)
C7—H7A	0.9600	N2—N3	1.383 (3)
C8—C9	1.512 (4)	N2—C8	1.296 (4)
C9—H9C	0.9600	N3—C10	1.423 (3)
C9—H9B	0.9600	N3—C5	1.359 (3)
C9—H9A	0.9600		
			
C1—C6—H6C	109.5	C12—C13—C14	119.9 (2)
C1—C6—H6B	109.5	C12—C11—H11	120.0
C1—C6—H6A	109.5	C12—C11—C10	119.9 (2)
C1—C2—H2	118.9	C13—C12—H12	119.5
C1—N1—C5	112.6 (2)	C13—C12—C11	121.0 (2)
C2—C1—C6	118.6 (2)	C13—C14—H14	119.9
C2—C3—C7	122.1 (2)	C13—C14—C15	120.2 (2)
C2—C3—C4	114.1 (2)	C14—C13—H13	120.0
C3—C7—H7C	109.5	C14—C15—H15	120.5
C3—C7—H7B	109.5	C14—C15—C10	119.0 (2)
C3—C7—H7A	109.5	C15—C14—H14	119.9
C3—C2—H2	118.9	C15—C10—N3	121.8 (2)
C3—C2—C1	122.1 (2)	H6A—C6—H6C	109.5
C3—C4—C8	136.4 (3)	H6A—C6—H6B	109.5
C4—C8—C9	130.0 (3)	H6B—C6—H6C	109.5
C4—C3—C7	123.8 (3)	H7A—C7—H7C	109.5
C5—C4—C8	104.1 (2)	H7A—C7—H7B	109.5
C5—C4—C3	119.5 (3)	H7B—C7—H7C	109.5
C5—N3—C10	131.7 (2)	H9A—C9—H9C	109.5
C5—N3—N2	109.0 (2)	H9A—C9—H9B	109.5
C8—C9—H9C	109.5	H9B—C9—H9C	109.5
C8—C9—H9B	109.5	N1—C1—C6	116.6 (2)
C8—C9—H9A	109.5	N1—C1—C2	124.8 (3)
C8—N2—N3	107.6 (2)	N1—C5—C4	126.9 (2)
C10—C11—H11	120.0	N2—C8—C9	119.0 (2)
C10—C15—H15	120.5	N2—C8—C4	111.0 (2)
C11—C12—H12	119.5	N2—N3—C10	119.3 (2)
C11—C10—N3	118.2 (2)	N3—C5—C4	108.2 (2)
C11—C10—C15	120.0 (2)	N3—C5—N1	124.8 (2)
C12—C13—H13	120.0		
